# Causal relationship between endometriosis with infertility and ankylosing spondylitis

**DOI:** 10.1038/s41598-023-40647-y

**Published:** 2023-08-17

**Authors:** Jian-xiong Wang, Yue Shen, Xi-le Wang, Lin-li Ma, Sheng-qian Xu

**Affiliations:** Department of Rheumatology & Immunology, The First Affiliated Hospital of Anhui Medical University, No. 218, Ji-xi Road, Hefei, 230022 Anhui China

**Keywords:** Spondyloarthritis, Risk factors

## Abstract

Retrospective studies have identified an increased risk of ankylosing spondylitis (AS) in endometriosis patients. The purpose of this study was to investigate the causal relationship between clinical phenotypes of endometriosis and AS using mendelian randomized analysis (MR). MR was performed using data from genome-wide association studies (GWASs). Heterogeneity, pleiotropy and sensitivity analyses were performed to evaluate the robustness of the results by MR Egger and inverse variance weighted (IVW), leave-one-out analysis. IVW, IVW-MRE (inverse variance weighted multiplicative random effects), weighted median and MR Egger were used to explore the relationship between endometriosis and AS. The IVW analysis showed a causal relationship between infertile endometriosis and AS (*OR* = 0.8334, *P* = 0.02191), and the same result was observed with IVW-MRE (*OR* = 0.8334, *P* = 0.0007933). However, further stratified analysis showed that no matter which statistical method was used, ovarian endometriosis (IVW: *OR* = 0.1662, *P* = 0.4986; IVW-MRE: *OR* = 0.1662, *P* = 0.4986; MR Egger: *OR* = − 0.9577, *P* = 0.2798; Weighted median: *OR* = 0.2628, *P* = 0.3452), pelvic peritoneum endometriosis (IVW: *OR* = 0.4363, *P* = 0.225; IVW-MRE: *OR* = 0.4363, *P* = 0.225, MR Egger: *OR* = 4.159, *P* = 0.1705; Weighted median: *OR* = 0.4112, *P* = 0.2714), rectovaginal endometriosis (IVW: *OR* = 0.1365, *P* = 0.805; IVW-MRE: *OR* = 0.1365, *P* = 0.805) there was no causal relationship between endometriosis and AS. This study suggested that patients with infertility endometriosis are at increased risk for AS. This study supports clinicians to pay more attention to the occurrence of AS in endometriosis patients with infertility.

## Introduction

Ankylosing spondylitis (AS) is a long-term inflammatory arthritis caused by autoimmune imbalance, affiliated to the spondyloarthritis (SpA). Its pathogenesis is related to immunological abnormalities involving tumor necrosis factor-α (TNF-α) and T helper17 cells. With the increasing understanding of the disease, gender problem of clinical is an increasing topical issue, the ratio of male to female in AS patients has been increased from 5:1 to 2:1 in a recent study, and 1:1 for nr-axSpA^1,2^. Therefore, more attention should be paid to the diagnosis and treatment of female patients with low back pain in the future.

Endometriosis is a familiar benign gynecological disease in which endometrial cells are active outside the uterine cavity. The increased levels of T helper17 cells subsets and TNF-α factor may provide an inflammatory environment for the occurrence and development of endometriosis^3^. According to a mutation analysis by whole exome sequencing, endometriosis was associated with multiple rheumatic immune diseases, including AS, systemic lupus erythematosus, and multiple sclerosis^4^. In 2022, a retrospective study firstly reported a higher risk of AS with endometriosis in Taiwan population^5^. However, the true causal relationship between endometriosis and the risk of AS may be influenced by various artificial subjective measurement biases and potential confounders in traditional epidemiology, leading to reverse causality. And conventional observational studies may also suffer from “residual confounding. Previous studies shown that endometriosis was only associated with some chronic inflammatory autoimmune diseases, for example rheumatoid arthritis, inflammatory bowel disease (IBD), excluding AS^6^.

Possible reasons for the association between endometriosis and an increased risk of developing AS are as follows: first, AS and endometriosis both have a strong genetic predisposition. Second, the two diseases may share various co-occurring genetic backgrounds. For example, there are variations in the expression levels of various genes associated with inflammation, angiogenesis, endothelial dysfunction, and immune disorders^7^. Therefore, it is worth exploring the causal relationship between the two diseases from multiple perspectives.

Mendelian randomization (MR) uses instrumental variables (IVs) in the analysis of genetic variation, to identify whether exposures and outcomes were observed and consistent with causal effects, avoiding potential confounders because genetic variants were randomly assigned^8^. Genome wide association studies (GWASs) database contain a large number of IVs, and accurate and reliable genetic interpretation of exposure by IVs can be obtained^9^. In addition, since single nucleotide polymorphisms (SNPs) occur before the onset of meiosis, the risk of reverse causality is minimized.

Therefore, the present study was designed to assess the causal effect of endometriosis in multiple clinical states on AS using multiple MR methods.

## Methods

### GWASs database

The instrumental variables(IVs) for endometriosis were downloaded from The GWAS summary data (https://gwas.mrcieu.ac.uk/). The affected site of endometriosis and the diagnosis of infertility were determined by a professional physician. Samples were taken for genetic testing to obtain GWAS data. All patient details are available on the gene data website (https://r6.finngen.fi/). Suitable genetic variants were selected from the GWAS database as effective IVs, including four endometriosis phenotypes (exposure IVs): endometriosis with infertility (ID: finn-b-N14_ENDOMET_INFERT), ovarian endometriosis (ID: finn-b-N14_ENDOMETRIOSIS_OVARY), pelvic peritoneal endometriosis (ID: finn-b-N14_ENDOMETRIOSIS_PELVICPERITONEUM), rectovaginal endometriosis (ID: finn-b-N14_ENDOMETRIOSIS_RECTPVAGSEPT_VAGINA). Outcome IVs was ankylosing spondylitis GWAS data (ID: Finn-b-M13_FORESTIER).

### IVs selection

Select IVs conditions:* P* < 5E−08, LD Rsq < 0.001, Clumping distance (kb) = 10,000. Each instrument of interest IVs and its proxies (r^2^ > 0.8). Palindromic IVs referred to the IVs with A/T or G/C alleles and "intermediate allele frequencies" referred to 0.01 < allele frequency < 0.30 were excluded from the above selected instrument IVs. F-Statistics for the IVs was solely calculated by the following equation: F = R2 (N − 2)/(1 − R2). R2 represented the variance of each collected IV. N was the sample size of original GWAS research, which were above the threshold of 10 as “strength instrument”. To calculate R2 for each IV, we used the following formula: R2 = 2β2EAF(1-EAF)/2β2EAF(1-EAF) + (se(β))2NEAF(1-EAF) where EAF was the effect allele frequency, beta was the estimated genetic effect on physical activity, N was the sample size of the GWAS and se was the standard error of the genetic effect. IVs with F statistics of less than ten were considered weak instruments and would be excluded for MR analysis for endometriosis with infertility, ovarian endometriosis, pelvic endometriosis and rectal endometriosis, respectively. Variables with the following diseases documented in the detailed GWAS data were also excluded: Inflammatory bowel disease^10^, vitamin D^11^, dyslipidemia^12^, physical activity^13^ and smoking^14^.

### MR assessment and analysis

The genetic variants for the IVs in the MR analysis will meet the following three assumptions: ① IVs must be closely linked to exposure; ② IVs were independent of any known confounders; ③ IVs must be associated with risk of outcome (AS) through exposure only. Nowadays, inverse variance weighted (IVW) analysis is regarded as the most accurate and reliable method for MR^15^, in the main analysis. Pleiotropy and heterogeneity assessment were estimated using MR Egger, IVW and Cochran’s Q statistic, respectively. A leave-one-out sensitivity analysis was performed to determine whether the associations were disproportionately affected by a single IVs. The scatter and Funnel plots from the MR analysis are used to visually compare this part of the results. IVW, inverse variance weighted-multiplicative random effects (IVW-MRE), weighted median and MR Egger were used to explore the causal relationship between endometriosis and the risk of AS. All analysis using R software environment (version 4.0.2) "TwoSampleMR" in the package."TwoSampleMR packages can get online:" https://mrcieulot.IO/TwoSampleMR.

## Results

Heterogeneity statistic analysed by MR Egger test and inverse variance weighted (IVW) test showed in supplementary 1. Horizontal pleiotropy analysis of each IVs were shown symmetry in funnel plots (supplementary 2). There was no heterogeneity in the results of this study. Statistical methods of IVW, IVW of multiplicative random effects (IVW-MRE) were used to analyse the causal relationship between ankylosing spondylitis and endometriosis as follows.

### Endometriosis

**Figure 1:**
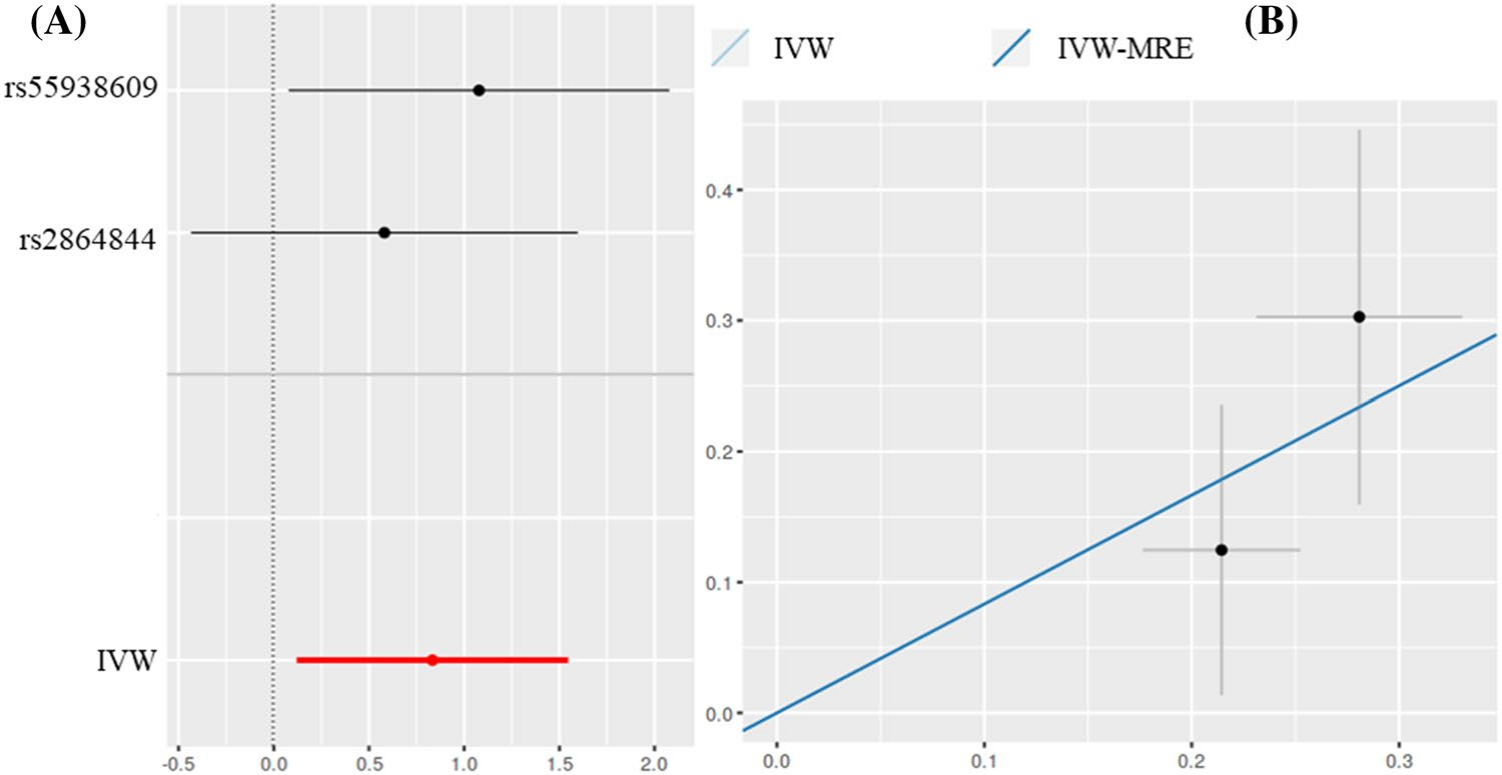
Scatter and forest plot of the association of infertile endometriosis with the risk of AS. (**A**) Forest plot: dots and lines indicate causal estimates of infertile endometriosis on the risk of AS. (**B**) Scatter plot: each black dot represents an SNPs plotted from the SNPs estimate on the endometriosis sign and the SNPs estimate on the risk of AS by the standard error bars. The slope of the line corresponds to the causal estimate using each of the different methods. The result of MR Egger was negative. Ankylosing spondylitis (AS), since single nucleotide polymorphisms (SNPs).

Using IVW examination, it was found that there was a risk causal relationship between infertile endometriosis and AS (*OR* = 0.8334, *P* = 0.02191). The risk of AS increased by 0.8334 times for each SD of endometriosis. The same results were also observed with IVW-MRE (*OR* = 0.8334, *P* = 0.0007933) (Fig. 1).

### Ovarian endometriosis

**Figure 2:**
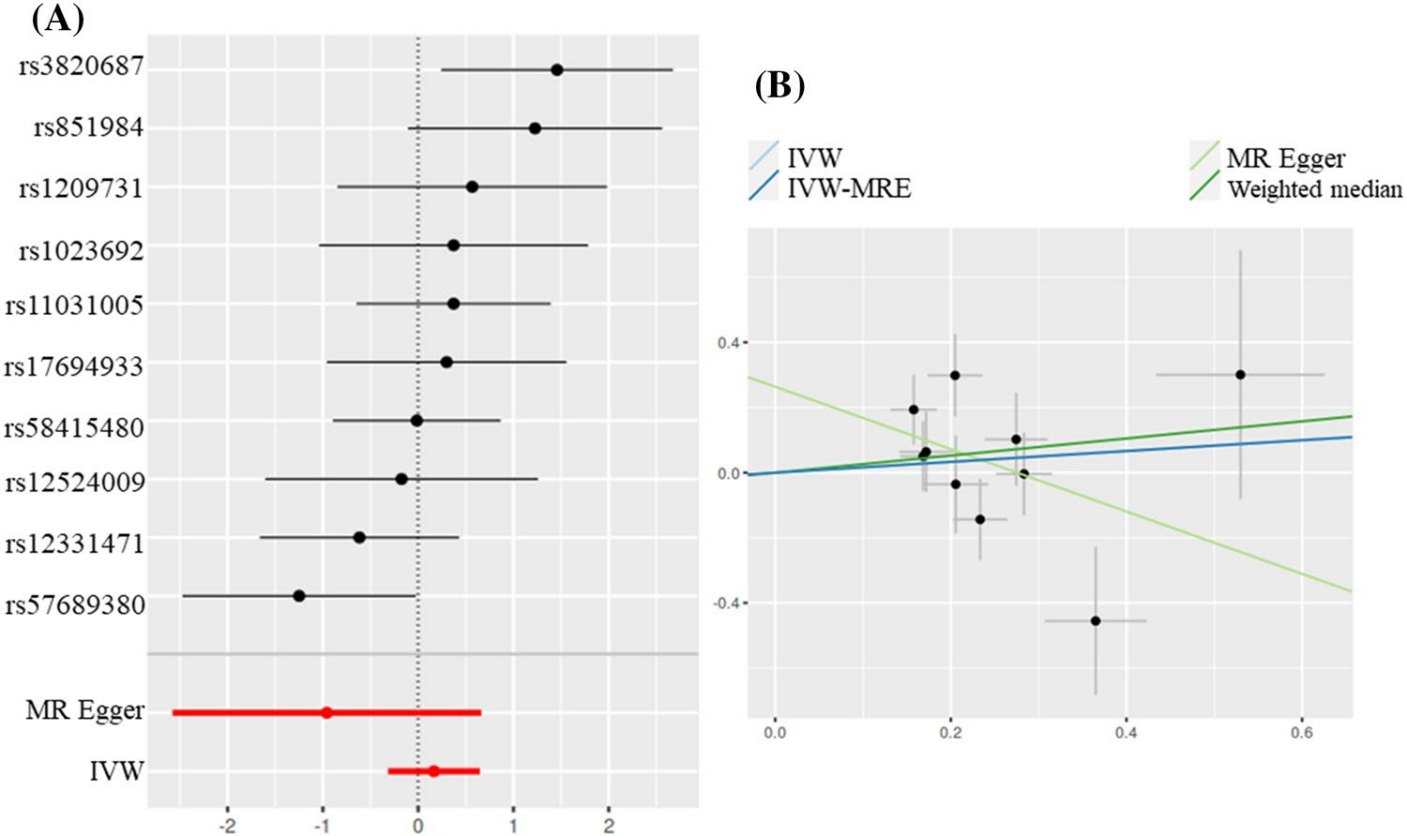
Scatter and forest plot of the association of endometriosis of ovary with the risk of AS. (**A**) Forest plot: dots and lines indicate causal estimates of ovarian endometriosis on the risk of AS. (**B**) Scatter plot: each black dot represents an SNPs plotted from the SNPs estimate on the ovarian endometriosis sign and the SNPs estimate on the risk of AS by the standard error bars. The slope of the line corresponds to the causal estimate using each of the different methods.

However, no causal relationship was found with AS in patients with endometriosis without infertility. Ovarian endometriosis and AS (IVW: *OR* = 0.1662, *P* = 0.4986; IVW-MRE: *OR* = 0.1662, *P* = 0.4986;MR Egger: *OR* = − 0.9577, *P* = 0.2798; Weighted median: *OR* = 0.2628, *P* = 0.3452) (Fig. 2).

### Pelvic peritoneal endometriosis

**Figure 3:**
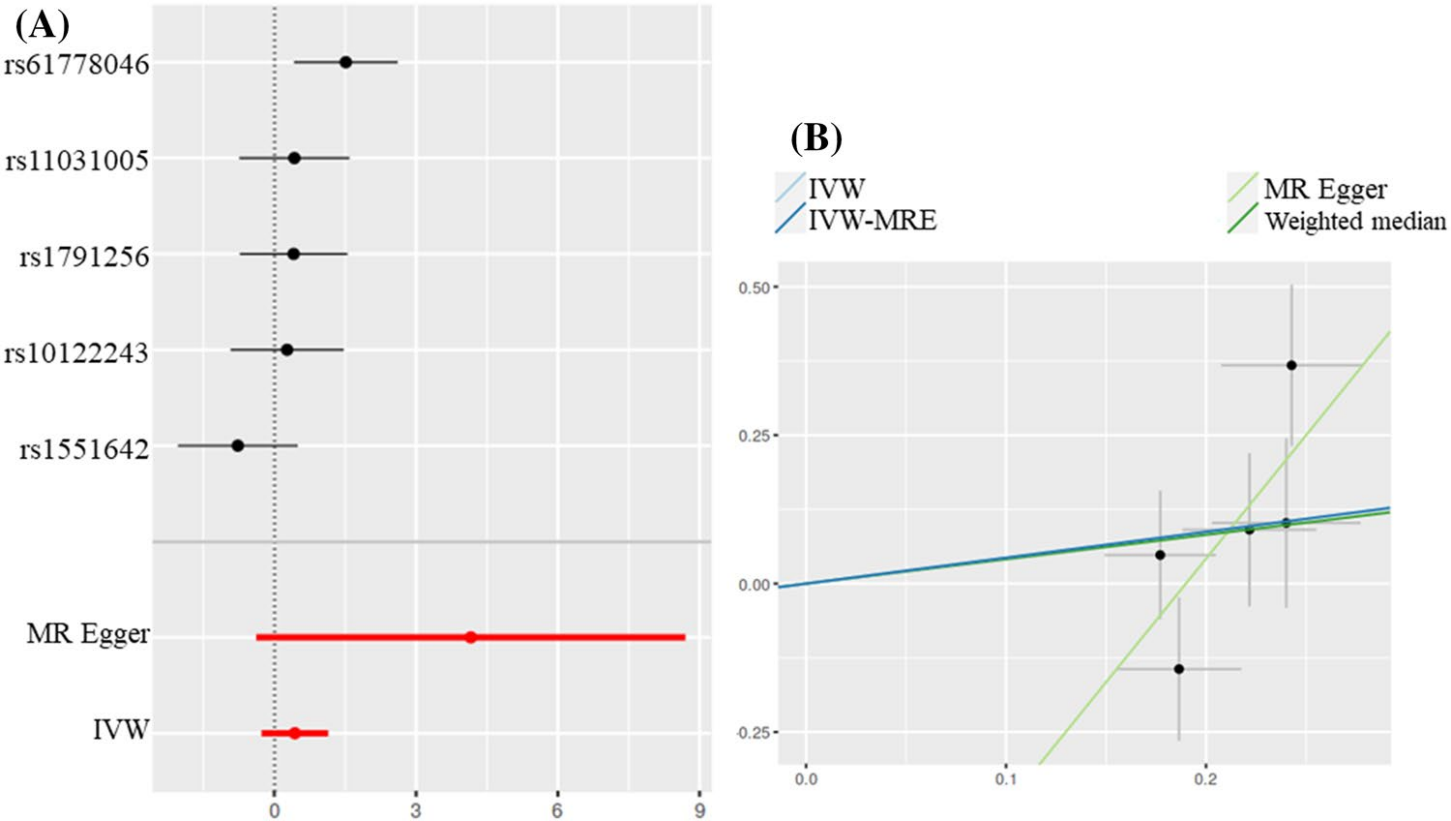
Scatter and forest plot of the association of endometriosis of pelvic peritoneal with the risk of AS. (**A**) Forest plot: dots and lines indicate causal estimates of pelvic peritoneal endometriosis on the risk of AS. (**B**) Scatter plot: each black dot represents an IVs plotted from the IVs estimate on the pelvic peritoneal endometriosis sign and the IVs estimate on the risk of AS by the standard error bars. The slope of the line corresponds to the causal estimate using each of the different methods.

Like ovarian endometriosis, pelvic peritoneal endometriosis don’t show statistically significant correlation with the risk of AS (Fig. 3). According to the IVW results, AS incidence increased 0.4363 times for each SD of pelvic peritoneal endometriosis (*P* = 0.225). Similar results were observed when various other statistical methods were used (MR Egger: *OR* = 4.159, *P* = 0.1705;Weighted median: *OR* = 0.4112, *P* = 0.2714).

### Rectovaginal endometriosis

**Figure 4:**
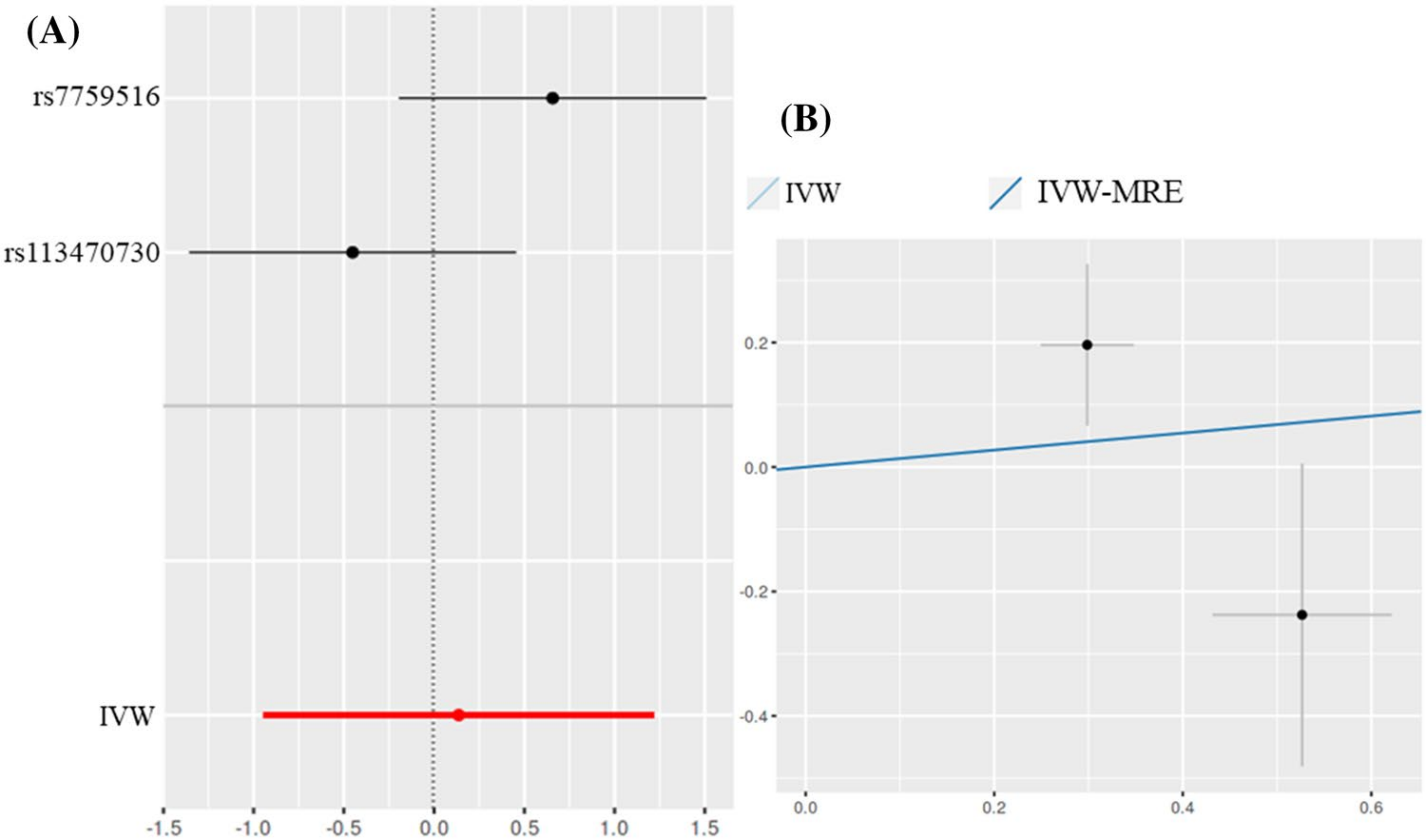
Scatter and forest plot of the association of endometriosis of rectovaginal with the risk of AS. (**A**) Forest plot: dots and lines indicate causal estimates of rectovaginal endometriosis on the risk of AS. (**B**) Scatter plot: each black dot represents an IVs plotted from the IVs estimate on the rectovaginal endometriosis sign and the IVs estimate on the risk of AS by the standard error bars. The slope of the line corresponds to the causal estimate using each of the different methods.

Rectovaginal endometriosis and AS (IVW: *OR* = 0.1365, *P* = 0.805; IVW-MRE: *OR* = 0.1365, *P* = 0.805) were not statistically significant (Fig. 4). Other statistical methods reached the same conclusion.

## Discussion

Nowadays, gender in clinical is an increasing topical issue, with the report of large sample cohort studies, the incidence of AS in women is gradually approaching in men^1,2^. It is a new challenge to pay attention to the diagnosis and treatment of AS in women, as endometriosis patients may show a higher risk of AS. In July 2022, Zhihua Yin et al.^5^ conducted a retrospective cohort study to investigate the correlation between endometriosis and AS. Cox-regression analysis showed that compared with the non-endometriosis cohort, patients with endometriosis had an increased risk of future AS incidence (*HR* = 1.61, 95%*CI* = 1.11–2.35, *P* = 0.013). Further comparison showed that the risk of AS was higher in patients aged 40 to 50 years with endometriosis than in other age groups (*HR* = 2.01, 95%CI = 1.15–3.49, *P* = 0.014). Therefore,hormonal changes in utero and chronic inflammation may play an important role in late-onset AS.

The MR analysis has a statistical advantage. Clinical studies are sometimes affected by confounding factors, for example smoking and menstruation.So, epidemiological studies may not be able to directly infer a causal relationship between AS and endometriosis. However, MR analysis can use SNPs as genetic variants to assess the causal relationship between exposure IVs and outcome IVs. Since genetic variation affects outcomes only through changes in exposure, they are randomly assigned and fixed before birth^16^. Therefore, the genetic evidence from this study can be a strong complement to the findings of previous observational clinical studies. In this study, information on genetic variants associated with AS and endometriosis was obtained from a large sample of GWAS data, and MR results shown that genetically predicted infertility endometriosis was causally associated with a significantly increased risk of AS (explore ID of Finn-B-N14_endomet_INFERT and outcome ID of Finn-b-M13_FORESTIER). The results were consistent with a case–control study published in July 2022^5^. The combination of the two studies basically confirms the close correlation between AS and endometriosis infertile.

The underlying mechanism triggering endometriosis and AS remains unclarified. Some studies have shown that endometriosis and AS may share the same genetic basis of variation, Matalliotaki et al.^4^ performed whole exome sequencing on endometriosis patients with third-generation family history. It was found that the patient was heterozygous for rs27434 and rs30187 IVs, T/C and A/G of ERAP1 gene, which was significantly related to the pathogenesis of AS. Solgi et al.^17^ found that people with elevated KIR2DS5 gene expression inhibit the development of endometriosis, AS and psoriasis. A case–control study was conducted to further compare the expression levels of KIR2DS5 in patients and controls.It was the protective factor of ankylosing spondylitis (*P* = 0.003, *OR* = 0.47, 95%*CI* = 0.28–0.79), endometriosis (*P* = 0.03, *OR* = 0.25, 95%*CI* = 0.07–0.82) and acute rejection of kidney Graft (*P* = 0.0056, *OR* = 0.44, 95%*CI* = 0.24–0.80)^18^. The tumor necrosis factor (TNF) family is one of the most critical factors in the occurrence and development of AS.The TNF family also significantly affects the gonadotropin-releasing hormone (GnRH) signal transduction mechanism, leading to gonadal hormone dysregulation through its influence on the thalamic-pituitary-adrenal/gonadal axis, and can lead to endometriosis^19^. Estrogen is known to modulate T cell differentiation and inhibit the differentiation of T helper17 cells^20,21^, T cells and T helper17 cells regulatory mechanism are also one of the important factors leading to AS. Previous reports have shown that estrogen levels are lower in patients with active AS than in patients with remitting^22^. It is also shown in mouse models, elevation of estrogen levels inhibited the development of arthritis^23^. These previous studies can partially explain the results of this paper. In this paper, we found that there is a causal relationship between AS in infertile endometriosis patients, and this outcome was not observed in non-infertile endometriosis patients, no significant causality was observed for other specific clinical subtypes (ovarian, pelvic peritoneum, rectovaginal). Infertility is often accompanied by premature changes in estrogen levels, which may contribute to endometriosis patients developing AS. Further studies are needed to elucidate the possible mechanisms by which genetic variants are involved in the association between endometriosis and AS.

There are limitations to this study. It should also have more AS-related IVs, and a larger sample size study would be more helpful for AS causality between endometriosis and provide more valid conclusions.

## Conclusion

In this Mendelian randomized study, we found an increased risk of AS in endometriosis patients with infertility. It is particularly important for clinicians to pay attention to the differential diagnosis of AS when assessing the low back range of motion in such patients and when presenting patients with low back pain.

### Supplementary Information

Supplementary Information 1.

Supplementary Information 2.

## Data Availability

The datasets for this study can be download in GWAS (GWAS ID included in the article), and further inquiries can be directed to the corresponding author.
