# Supplementary material for: Causal relationship between endometriosis with infertility and ankylosing spondylitis
Source: Sci Rep. 2023 Aug 17;13:13412. doi: 10.1038/s41598-023-40647-y (PMC10435539; doi:10.1038/s41598-023-40647-y)
Supplement: Supplementary file 1 — Supplementary Information 1. [file 41598_2023_40647_MOESM1_ESM.pdf]

Supplementary 1. Heterogeneity analysis

Outcome	Exposure	Method	Q	Q_df	P-value
Ankylosing spondylitis, ID: Finn-b-M13_FORESTIER	Endometriosis with infertility, ID: finn-b-N14_ENDOMET_INFERT	MR Egger	-	-	-
		IVW	0.4667	1	0.4945
	Ovarian endometriosis, ID: finn-b-N14_ENDOMETRIOSIS_OVARY	MR Egger	12.00	8	0.1513
		IVW	15.01	9	0.09063
	Pelvic peritoneal endometriosis, ID: finn-b-N14_ENDOMETRIOSIS_PELVICPERITONEUM	MR Egger	3.871	3	0.2758
		IVW	7.26	4	0.1228
	Rectovaginal endometriosis, ID: finn-b-N14_ENDOMETRIOSIS_RECTPVAGSEPT_VAGINA	MR Egger	-	-	-
		IVW	3.048	1	0.08081

IVW: Inverse variance weighted
