# Supplementary figures and images for: Causal relationship between endometriosis with infertility and ankylosing spondylitis
Source: Sci Rep. 2023 Aug 17;13:13412. doi: 10.1038/s41598-023-40647-y (PMC10435539; doi:10.1038/s41598-023-40647-y)

## Supplementary 2. Funnal plot of horizontal pleiotropy analysis

(a)

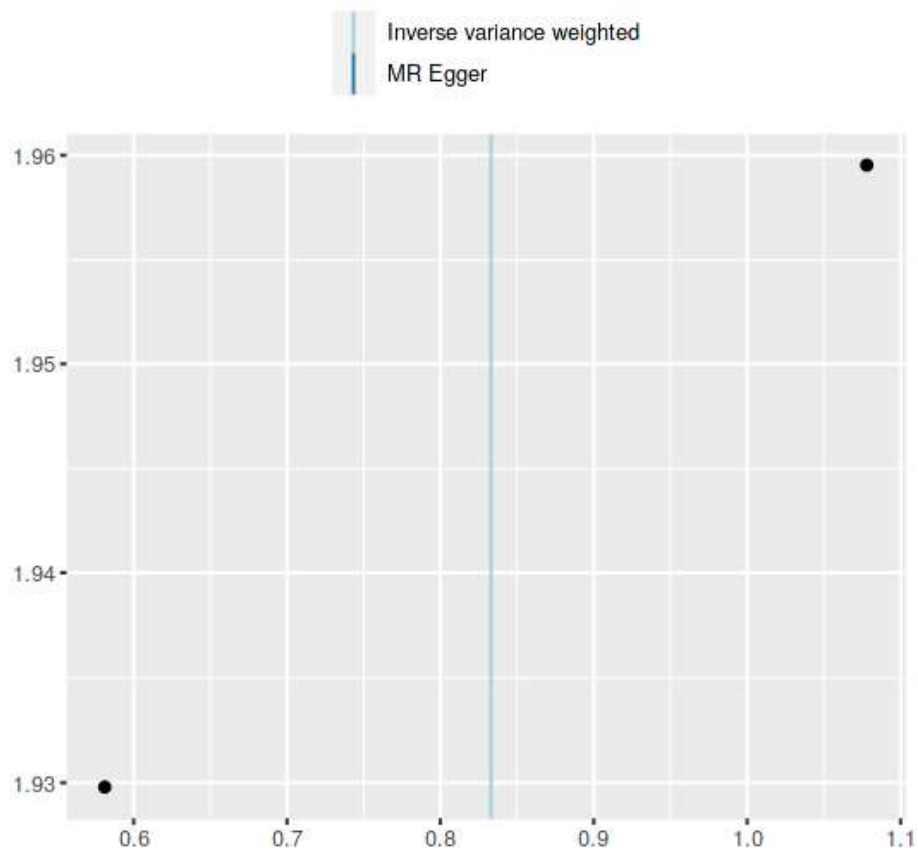

(b)

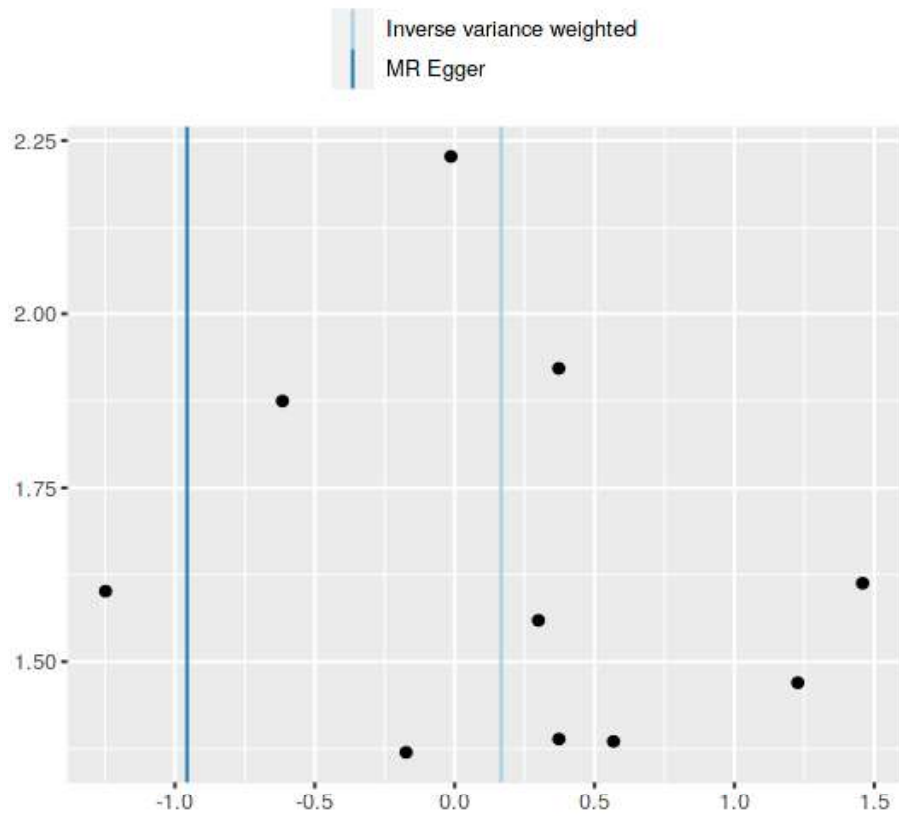

(c)

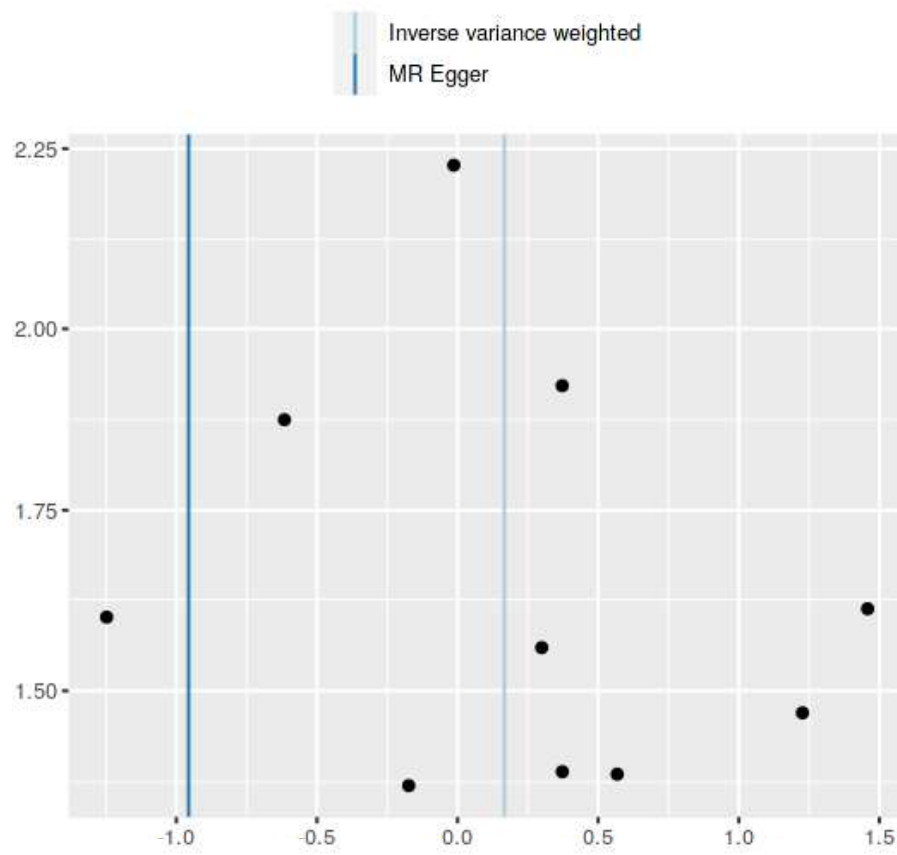

(d)

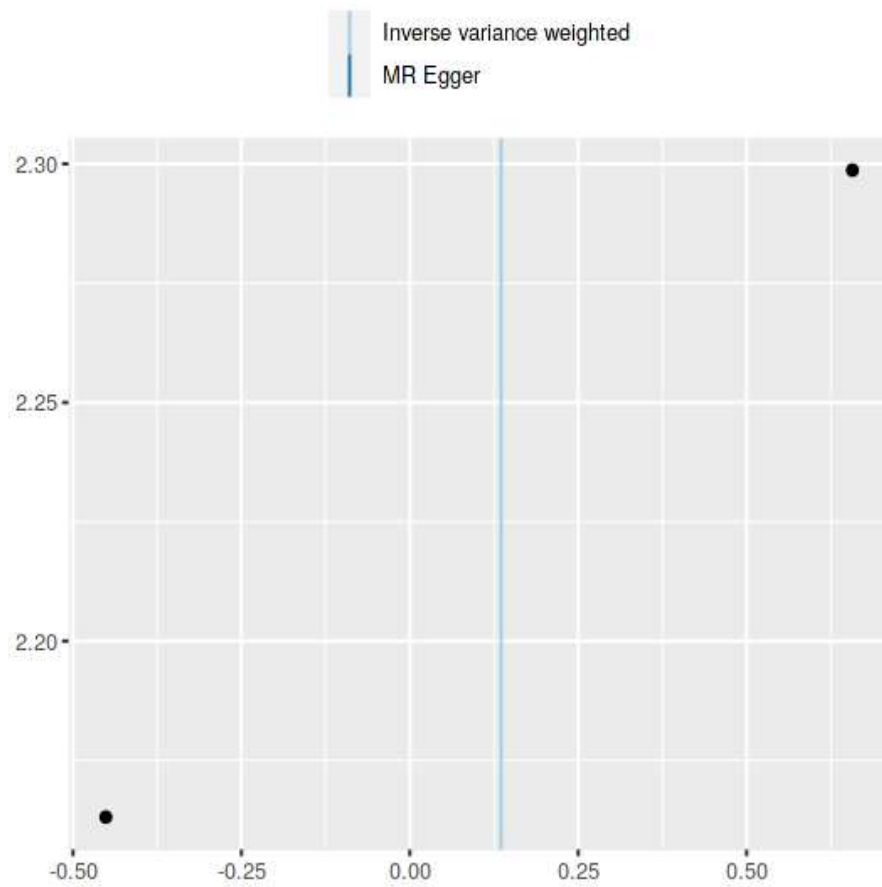

Supplement: Supplementary file 2 — Supplementary Information 2. [file 41598_2023_40647_MOESM2_ESM.pdf]
